# Photosynthetic Responses of Canola to Exogenous Application or Endogenous Overproduction of 5-Aminolevulinic Acid (ALA) under Various Nitrogen Levels

**DOI:** 10.3390/plants9111419

**Published:** 2020-10-23

**Authors:** Xinxin Feng, Yuyan An, Jingjing Gao, Liangju Wang

**Affiliations:** 1 College of Horticulture, Shanxi Agricultural University, Taigu 030801, China; fengxx@sxau.edu.cn (X.F.); gaojj@sxau.edu.cn (J.G.); 2 College of Horticulture, Nanjing Agricultural University, Nanjing 210095, China; anyuyan0447@njau.edu.cn

**Keywords:** 5-aminolevulinic acid (ALA), photosynthetic responses, nitrogen supply, canola

## Abstract

Limited data are available on the effects of 5-aminolevulinic acid (ALA) on plant photosynthesis in relation to the nitrogen (N) level. In this study, we investigate photosynthetic responses to ALA in canola plants (*Brassica napus* L.). We used wild-type plants without ALA addition (controls), wild-type plants with exogenous ALA application, and transgenic plants that endogenously overproduced ALA. The plants were grown hydroponically in nutrient solutions with low, middle, and high concentrations of N. Our results indicate that plants in both treatment groups had higher chlorophyll contents and net photosynthetic rates and lower intracellular CO_2_ concentrations in the leaves, as compared to controls. Furthermore, simultaneous measurement of prompt chlorophyll fluorescence and modulated 820-nm reflections showed that the active photosystem II (PS II) reaction centers, electron transfer capacity, and photosystem I (PS I) activity were all higher in treated plants than controls at all N levels; however, the responses of some photochemical processes to ALA were significantly affected by the N level. For example, under low N conditions only, a negative ΔK peak appeared in the prompt chlorophyll fluorescence curve, indicating a protective effect of ALA on electron donation via activation of the oxygen-evolving complex. Taken together, our findings suggest that ALA contributes to the promotion of photosynthesis by regulating photosynthetic electron transport under various N levels. These findings may provide a new strategy for improving photosynthesis in crops grown in N-poor conditions or reduced N-fertilization requirements.

## 1. Introduction

In plants, photosynthesis serves as the foundation for all metabolic processes and is thus considered to be one of the most susceptible physiological processes to biotic and abiotic stresses [1,2]. This process is usually divided into photoreaction and dark reaction. In photochemical reactions, photosystem II (PS II) uses light energy to strip electrons from water and release O_2_. Then, electrons are transferred to photosystem I (PS I) via the plastoquinone (PQ) pool, cytochrome b_6_f complex, and plastocyanin (PC) to produce nicotinamide adenine dinucleotide phosphate (NADPH). Meanwhile, the electron transfer reaction also produces a proton gradient on the thylakoid membrane, followed by the production of adenosine triphosphate (ATP) through ATP synthetase. Eventually, both ATP and NADPH provide fuel for the Calvin cycle that holds carbon dioxide in a dark reaction.

Nitrogen (N) is an essential element for plants, which plays an important role in increasing crop yield and improving the quality of agricultural products [3,4]. N deficiency leads to a decrease in leaf photosynthetic rate, resulting in a decrease in yield [5]. When N supply is insufficient, the decrease in the photosynthetic rate of plants is caused by many factors, including a decrease in stomatal conductance [6], pigment degradation [7], and a decrease in the light and dark reaction ability of photosynthesis [8,9]; however, increasing agricultural yield by applying a large amount of N fertilizer is not advisable. Excessive application of N often comes with environmental costs: water pollution, soil degradation, trace gas emission, climate changes, and loss of biodiversity [10]; therefore, a strategy to maintain an appropriate rate of photosynthesis under low N conditions without increasing more N input is required.

The application of bioregulators (plant growth regulators and endogenous plant hormones) to plants is an effective way to enhance photosynthesis. 5-Aminolevulinic acid (ALA) is an essential precursor to tetrapyrrole biosynthesis in plants, and it plays key roles in various physiological and biochemical processes, including heme and chlorophyll (Chl) biosynthesis [11], hormonal activities [12,13], resistance to various stresses [14,15,16], and fruit coloration [17,18]. The important role of ALA in photosynthesis was revealed by applying it exogenously to plants growing under normal [19,20] and stressful conditions [21,22,23,24] and through the study of endogenous ALA-overproducing transgenic plants [25,26]. These studies suggest that the proposed mechanisms underlying photosynthesis improvement by ALA might be related to increases in Chl content [21], photosynthetic electron transport activity [22], antioxidant activity [23,24], and Rubisco activity [25]; however, none of those studies have evaluated photosynthetic responses in ALA-treated plants grown under various levels of N availability. Some studies have investigated the role of ALA in N metabolism and suggested that it may promote N absorption and assimilation [27,28]. This led us to hypothesize that the N supply might play an important role in the physiology of endogenous ALA-overproducing transgenic plants and the effects of exogenous ALA application.

Analysis of prompt Chl fluorescence (PF, also called OJIP) curves can allow for the examination of the effects of ALA on the behavior of PS II under stress. This is because PF is a reliable and sensitive measurement method that can provide a great deal of useful information on the photosynthetic apparatus [29,30]. In a study of watermelons, Sun et al. [24] found that ALA increased the possibility that a trapped exciton would move an electron into the electron transport chain beyond Q_A_^–^ (Ψ_Eo_) and the quantum yield of electron transport (φ_Eo_) but decreased the closure rate of active reaction centers Q_A_ (M_o_). Recently, An et al. [31] observed that ALA markedly improved photosynthetic performance indexes (PI_ABS_ and PI_total_) and significantly reduced M_o_, the amount of dissipated energy (DI_o_/RC and DI_o_/CS), and the relative variable fluorescence at J-step (V_J_) in fig plants under waterlogging stress. In addition, electron transport after PQ and to the PS I acceptors can be detected by the modulated reflection (MR) signal measured at 820 nm [32,33]. In recent years, a new instrument that simultaneously measures PF and MR signals has been developed to explore the photosynthetic electron transport process and the interaction between PS II and PS I in plant leaves [34,35]. The effects of ALA on the redox states of PS II and PS I reaction centers were recently reported in strawberry leaves by analyzing PF and MR signals [20]. Unfortunately, very limited data are available on the effects of ALA on PF and MR curves and the related fluorescence parameters under different nutritional environments.

In the present study, we investigate the effects of ALA on photosynthetic responses under various N conditions in terms of Chl content, gas exchange parameters, and PF and MR curves. We examined the effects of exogenous ALA on photosynthesis-related parameters in wild-type (WT) canola and compared them with the photosynthetic responses of an ALA-overproducing transgenic line (T). The objective of this study is to determine the influences of ALA on photosynthesis-related parameters and PS I and PS II components in canola leaves under various levels of N nutrition.

## 2. Results

### 2.1. Chl Content 

**Figure 1:**
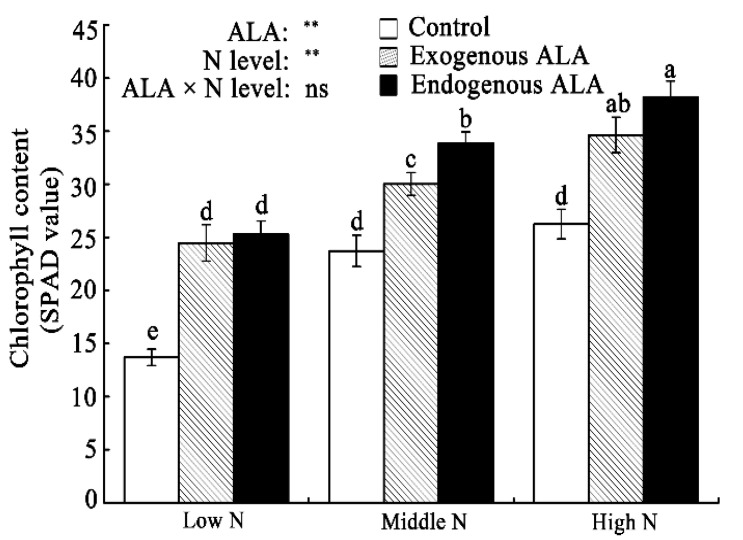
Differences in chlorophyll content (represented as SPAD values) in exogenously 5-aminolevulinic acid (ALA)-treated and endogenous ALA-overproducing canola leaves grown under low-, middle-, and high-N conditions. Means ± standard errors are presented (*n* = 10). Means with the same letter indicate a nonsignificant difference (*p* > 0.05), according to Duncan’s test. Sources of variation: ALA, N-level, and ALA × N-level interaction; ** *p* < 0.01; ns: not significant. N = nitrogen.

Both N levels and ALA presence markedly influenced the Chl content (represented as SPAD values) of canola leaves (Figure 1). The mean Chl contents of plants grown under middle- and high-N conditions were 38% and 56% higher, respectively, than those plants grown under low-N conditions. This indicates that canola is a nitrophilous plant and that a higher NO_3_^-^ concentration in the culture media results in higher Chl contents in the leaves. Compared with controls, exogenous and endogenous ALA enhanced the Chl contents of canola leaves grown under all N levels. There were no significant interactions between the effects of ALA presence and N level on Chl content.

### 2.2. Photosynthetic Gas Exchange Characteristics

To further investigate the effects of exogenous and endogenous ALA on photosynthesis in canola plants grown under various N levels, we determined gas-exchange parameters, including the net photosynthetic rate (Pn), intercellular CO_2_ concentration (Ci), stomatal conductance (Gs), and transpiration rate (Tr).

**Figure 2:**
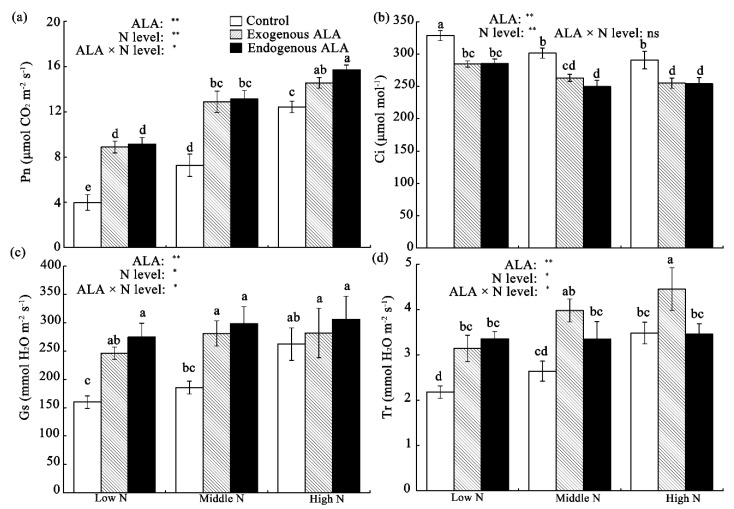
Differences in (**a**) net photosynthetic rate (Pn), (**b**) intercellular CO_2_ concentration (Ci), (**c**) stomatal conductance (Gs), and (**d**) transpiration rate (Tr) in exogenously-treated and endogenous ALA-overproducing canola leaves grown under low-, middle-, and high-N conditions. Values are means ± standard errors (*n* = 10). The same letter indicates no significant difference (*p* > 0.05) according to Duncan’s test. Sources of variation: ALA, N-level, and ALA × N-level interaction; * *p* < 0.05; ** *p* < 0.01; ns: not significant.

In the control plants, when the N levels increased, the Pn, Gs, and Tr levels increased, while the Ci levels decreased. Compared with controls, both exogenous and endogenous ALA plants had increased Pn but decreased Ci at all N levels. Increased Gs was also observed in the leaves of both the low- and middle-N groups. Exogenous ALA plants had increased Tr at all N levels, but endogenous ALA-overproducing plants did not have altered Tr under the middle- and high-N conditions. Interactions between ALA and N levels were observed for three of the parameters but not Ci (Figure 2). 

Correlation analysis showed that Pn was positively related to Chl content, Gs, and Tr, and negatively related to Ci (Table S1).

### 2.3. PF Curves and JIP-Test

**Figure 3:**
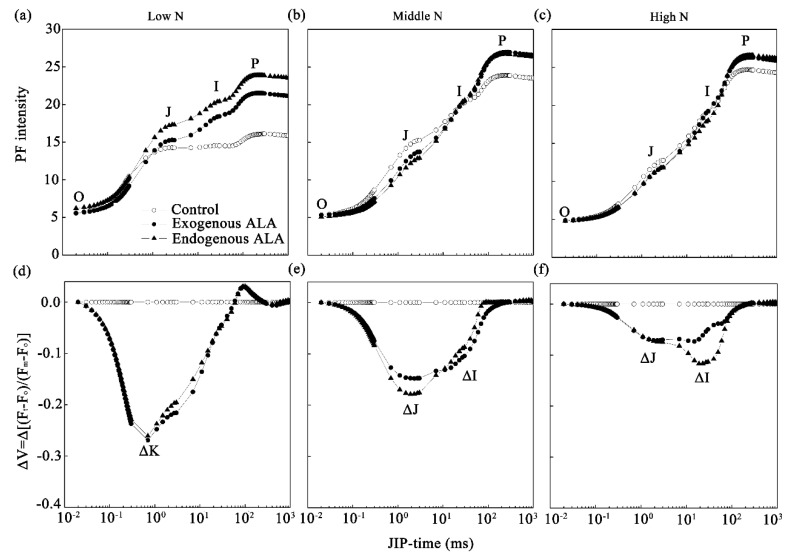
Changes in prompt fluorescence (PF) curves in exogenously-treated and endogenous ALA-overproducing canola leaves under low- (**a**,**d**), middle- (**b**,**e**), and high-N conditions (**c**,**f**). (**a**–**c**) PF curves plotted on a logarithmic time scale from 20 μs to 1 s (JIP time). The steps O (at 20 µs), J (at 2 ms), I (at 30 ms), and P (peak) are marked. Each curve is the average of 10 replicate measurements. (**d**–**f**) Variable fluorescence curves (ΔV = Δ[(F_t_ − F_o_)/(F_m_ − F_o_)]), which were constructed by subtracting the normalized (between the O step and P step) values of the PF recorded in wild-type (WT) canola. The feature peaks ΔK, ΔJ, and ΔI are marked.

Separation of the PF curves (OJIP curves; Figure 3a–c) facilitates the distinction of the various effects of ALA at each N level. This shows that the kinetic curve of PF induction of canola leaves is very sensitive to N supply. With the low-N solution, the I and P steps of the exogenous ALA-treated canola were markedly higher than those of controls and were even higher in the transgenic plants. For plants supplied with the middle-N solution, the J steps in the ALA treatments were significantly lower than those of controls, while the P steps were higher. However, as shown in Figure 3c, with the high-N solution, the P steps in the exogenous ALA-treated and endogenous ALA-overproducing canola were still significantly higher than those of controls. Thus, the amplitude of the I-to-P phase was always observed to be higher in the exogenous ALA-treated and endogenous ALA-overproducing canola than in controls at all N levels. 

The PF curves were normalized from the O step to the P step and presented as the relative variable fluorescence (Figure 3d–f). We found that a sharp V-shaped curve with a negative ΔK peak appeared at 0.7 ms under the low-N condition, and a W-shaped curve appeared with two negative peaks (ΔJ at approximately 2 ms and ΔI at approximately 20 ms) under the middle- and high-N conditions. We also compared the effects of N level on canola leaves (Figure S1). The results show that, compared with the high-N condition, a ΔK peak appeared under the low-N condition and ΔJ and ΔI appeared under the middle-N condition. Thus, the occurrence of these feature peaks was dependent on the N supply.

**Figure 4:**
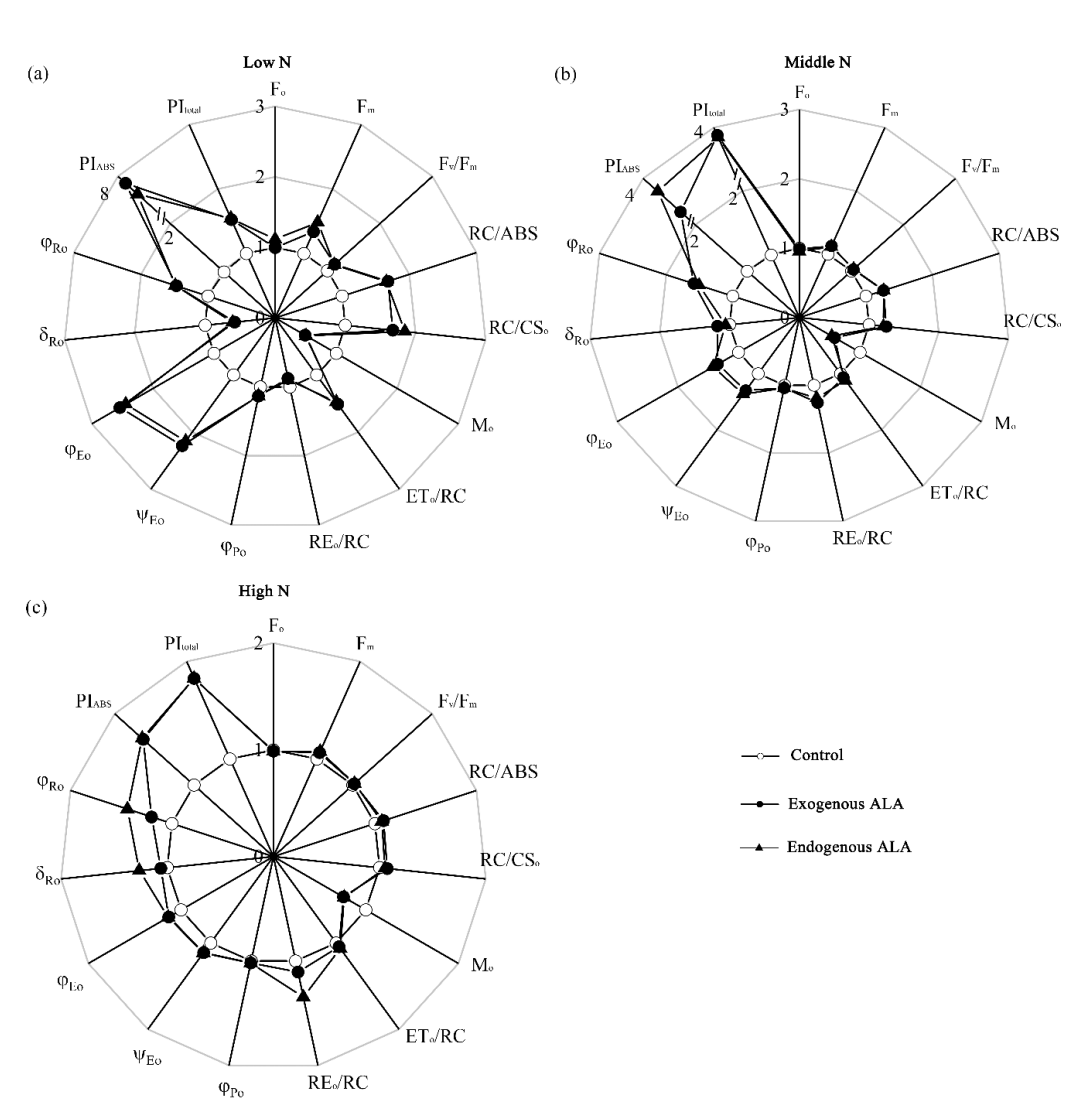
Radar plots of changes in JIP test parameters in exogenously treated and endogenous ALA-overproducing canola leaves relative to controls under (**a**) low-, (**b**) middle-, and (**c**) high-N conditions.

The variation in the selected parameters derived from the PF curves is shown in Figure 4 and Table S2. In the radar plots (Figure 4), the initial values of the parameters in the controls were scaled to 1, and the parameters in the treatment groups were calculated as ratios of the initial values. Exogenous and endogenous ALA induced increases in the maximum yield of primary photochemistry of PS II (F_v_/F_m_), Q_A_-reducing reaction centers per PS II antenna Chl (RC/ABS), the quantum yields and efficiencies/probabilities (φ_Po_, ψ_Eo_, φ_Eo_, and φ_Ro_), specific energy fluxes per one PS II reaction center (ET_o_/RC), performance indexes (PI_ABS_ and PI_total_), and phenomenological energy flux per excited cross-section values (RC/CSo). There was a decrease in M_o_ at all N levels, but this did not alter F_o_. In the exogenous ALA-treated and endogenous ALA-overproducing canola, δ_Ro_ and RE_o_/RC increased under the middle- and high-N conditions, especially under the former condition. Overall, ALA-induced changes in PF parameters might become less pronounced with increasing N levels. 

Furthermore, leaf Pn was negatively related to M_o_ but was positively related to F_m_, F_v_/F_m_, ET_o_/RC, φ_Po_, ψ_Eo_, φ_Eo_, φ_Ro_, RC/ABS, PI_ABS_, and PI_total_ (Table S1). 

### 2.4. MR Curves and Related Parameters

**Figure 5:**
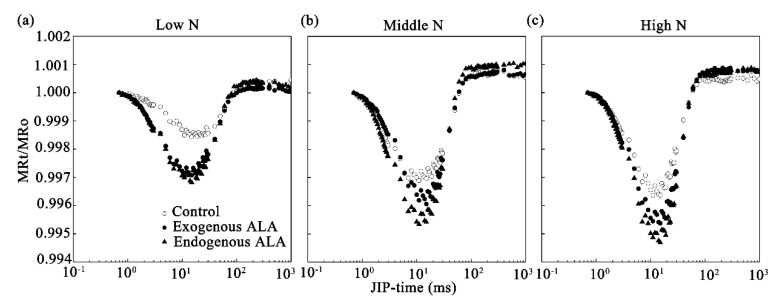
Changes in 820-nm modulated reflection (MR) curves, expressed as normalized MR (represented as MR_t_/MRo), in exogenously-treated or endogenous ALA-overproducing canola leaves grown under (**a**) low-, (**b**) middle-, and (**c**) high-N conditions.

Figure 5 shows the changes in the normalized MR (represented as MR_t_/MR_o_) induced by red actinic light in canola leaves in response to exogenous and endogenous ALA treatment. Kinetic changes in MR reflect the redox states of P700 and PC, i.e., initial oxidation of P700 and PC was followed by rereduction when electrons arrive from PS II [25]. The minimal MR_t_/MR_o_ (MR_min_) value indicates that the oxidation and rereduction rates of P700 and PC were equal and that MR_min_ was significantly decreased by the low N supply in WT canola. A lower MR_min_ was always observed in the ALA treatment groups. The ALA-induced change in MR_min_ did not depend on the N level, suggesting that ALA treatment generally motivates electrons from P700 and the PC pool through the linear electron transfer chain to reduce end-acceptors such as nicotinamide adenine dinucleotide phosphate and form NADPH, which is necessary for CO_2_ fixation.

**Figure 6:**
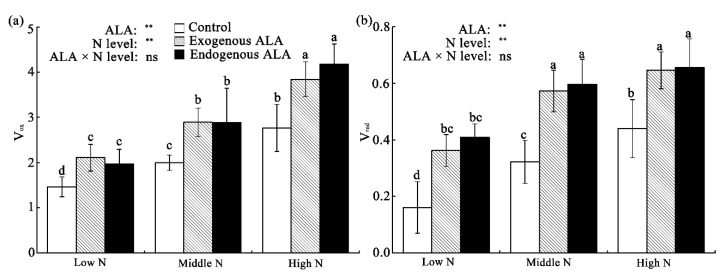
Changes in the velocities of (**a**) P700 and plastocyanin (PC) oxidation (V_ox_) and (**b**) subsequent rereduction (V_red_) in exogenously-treated and endogenous ALA-overproducing canola leaves grown under low-, middle-, and high-N conditions. Values are means ± standard error (*n* = 10). The same letter indicates a nonsignificant difference (*p* > 0.05), according to Duncan’s test. Source of variation: ALA, N-level, and ALA × N-level interaction; ** *p* < 0.01; ns: not significant.

The velocities of P700 and PC oxidation (V_ox_) and subsequent rereduction (V_red_) can be calculated from the maximal slopes of the kinetic curves of photoinduced MR changes (Figure 6). In the controls, V_ox_ and V_red_ gradually increased with increases in the N level. Compared with controls, V_ox_ and V_red_ were significantly higher in the ALA treatment plants. There were no significant ALA or N-level interaction effects on V_ox_ and V_red_. 

Furthermore, leaf Pn was positively related to V_ox_ and V_red_ (Table S1).

## 3. Discussion

Applying N markedly affected the photosynthetic ability of canola leaves [36,37]. The data from this experiment support previous observations and show that increases in Chl content, Pn, Gs, and Tr, and decreases in Ci, are associated with increasing N levels (Figure 1 and Figure 2). Furthermore, we found that WT canola grown under low-N conditions exhibited dramatically depressed J and P steps in the PF curve (Figure S1), which indicates that electron flow from the reduced Q_A_ to Q_B_ was inhibited under low N conditions. Similar findings have been reported for N-deficient radishes and algae [38,39] and pea plants under high-temperature stress [40]. In this study, the low N level caused a general restriction of electron transport from PS II to PS I in WT canola leaves. This is also supported by our findings that show that the maximum PS II efficiency of dark-adapted leaves (F_v_/F_m_) and the performance indexes (PI_ABS_ and PI_total_) were suppressed under the low-N condition (Table S2).

Improved photosynthesis after exogenous application of ALA was reported under normal conditions [19] and various stressful conditions, such as cold [41], salinity [23,42], low light [22,43], waterlogging [31], and heat stress [44]. Thus, we can conclude that ALA can promote plant photosynthesis under multiple conditions. The results of this study also confirm that ALA can improve photosynthesis in canola plants grown with various amounts of N, suggesting that ALA has great application potential in agriculture. In addition, we used a transgenic line of canola that contained a constituted gene *YHem1*, which biosynthesized more ALA than the plant would otherwise produce; we found that this plant exhibited improved photosynthesis [45]. Sun et al. [25] suggested that, when transformed into canola, the *YHem1* gene accelerates endogenous ALA metabolism, leading to greater Chl accumulation, higher diurnal photosynthetic rates, and upregulated expression of the gene that encodes the Rubisco small subunit. Thus, both exogenous and endogenous ALA can be used as models to study the mechanism of ALA-reinforced photosynthesis.

The photosynthetic rate (Pn) is generally higher in plants treated exogenously with ALA and those that endogenously overproduce ALA [20,25]. The negative relationship between leaf Pn and Ci (Table S1) in this study suggests that the ALA-induced increases in leaf Pn that were observed at all N levels (Figure 3a) were mainly associated with nonstomatal factors, as was reported in watermelon [24]. Although ALA is the key precursor in Chl biosynthesis [11,12], it is difficult to attribute ALA’s promotion of plant photosynthesis to greater accumulation of Chl. Hotta et al. [46] suggested that the accumulation of Chl in plants can be stimulated by treatment with ALA alone; however, using ALA to promote photosynthesis also requires a combined nutrient supply. Furthermore, Liu et al. [47] found that exogenous ALA treatment did not influence Chl content but enhanced the photosynthetic rate in strawberry leaves. Thus, other mechanisms involved in the regulation of Chl biosynthesis may be involved in photosynthesis when plants are treated with ALA. In fact, in addition to Chl content, exogenous or endogenous ALA also affects the Chl a/b ratio [25,48], which is related to the antenna size of PS I and PS II reaction centers [49]; therefore, the ratio of PS II/PS I may be regulated by ALA.

The promotion of photosynthetic capacity by ALA could be related to its effect on the electron transport chain. We observed that ALA-treated plants had more active PS II reaction centers (estimated as an increase in RC/ABS) than untreated plants at all N levels. The higher RC/ABS implies that either the active PS II reaction centers were more numerous or the apparent antenna size was smaller [38,50]. In this study, the increase in active PS II reaction centers in ALA-treated plants was supported by an increase in the active reaction centers per excited cross-section (RC/CSo) and a decrease in the closure rate of reaction centers (Mo). Additionally, we found that ALA enhanced the efficiency of electron transfer from PS II to the acceptor side of PS I in the intersystem chain, characterized by an increase in φ_Po_, ψ_Eo_, φ_Eo_, and φ_Ro_ in the ALA-treated plants. 

Regardless of N level, ALA-treated plants had greater PS I activity than untreated plants. Until now, the reason why ALA induces an increase in the IP phase was unclear. Many reports have shown that the IP phase follows the rereduction of PC^+^ and P700^+^, indicating that this phase might be primarily related to PS I redox [38,40]. This is also supported by our findings: IP, along with V_red_, was higher in the leaves of both treatments than in controls (Figure 3 and Figure 6b). Additionally, considering that the electron transport efficiency at the PS I end-electron acceptors may represent the size of the PS I reaction centers, the amplitude of the IP phase also reflects changes in PS I content [51]. High PS I activity may endow ALA-treated plants with a high capacity for physiological adaptation in response to various stressful conditions. This agrees with the findings of Sun et al. [24], who reported that ALA affects PS I reaction centers by promoting antioxidant enzyme activity. This can cause the scavenging of superoxide anions around PS I, leading to an increase in the apparent electron transport rate. Furthermore, the parameters φ_Ro_, δ_Ro_, RE_o_/RC, and PI_total_ were related to PS I activity; however, in our study, at all N levels, these four parameters were affected differently according to treatment type (Figure 4). The lower δ_Ro_ and RE_o_/RC in the ALA treatment groups were only evident under the low-N condition. This could be explained by the results of Kalaji et al. [52], who found that changes in δ_Ro_ and RE_o_/RC are sensitive to nutrient deficiency. 

ALA-treated plants exhibited N-dosage-specific effects on PS II activity, as indicated by the appearance of three feature peaks at all N levels (Figure 3d–f). The ΔK peak is an important feature of serious nutrient deficiency, as reported in radishes [38], citrus [50], maize and tomatoes [52], and beans [53]. The appearance of a positive ΔK peak suggests that the oxygen-evolving complex (especially the Mn complex on the PS II donor side) was inactivated and the antenna complexes were more connected, possibly due to improper membrane organisms. Thus, there was lower energy transfer and absorption efficiency in those leaves, especially the N-deficient ones [50]. On the contrary, a negative ΔK peak occurred in both treatment groups under the low-N condition (Figure 3d), confirming earlier observations that ALA promotes activity on the donor side of PS II reaction centers [24]. Further, ΔJ and ΔI peaks are associated with reductions in Q_A_ and the plastoquinone pool at the PS II acceptor side, respectively [54,55]. Positive ΔJ and ΔI bands have been observed in moderately-nutrient-deficient plants, such as maize and tomatoes [52], and N-deficient radishes [38]. In the present study, the appearance of negative ΔJ and ΔI peaks (Figure 3e,f) suggests that ALA increased the activity of the PS II acceptor side under the middle- and high-N conditions. This suggests that there are two types of ALA responses according to the N level. One type corresponds with damage repair under low-N conditions, as indicated by the parameter ΔK. The other type is reflected in enhanced performance indexes and electron transport efficiencies under middle- and high-N conditions. 

## 4. Materials and Methods

### 4.1. Plant Materials and Growth Conditions

The canola (*Brassica napus* L.) used in this study comprised a wild-type (WT) and an ALA-overproducing transgenic line (T). The T canola contains a recombinant gene *YHem1*, i.e., yeast *Hem1* (aminolevulinate synthase-coded gene), controlled by a light-responsive promoter of the *HemA1* gene from *Arabidopsis thaliana* [26,45]. Thus, the transgenic plants synthesized more endogenous ALA under light because of additional *YHem1* expression and aminolevulinate synthase activity, so that the ALA content in the transgenic plants was significantly higher than that in the WT [25,45]. The transgenic canola seeds that were used in this study were at generation 5 and were homogenous.

We carried out the experiment in a plastic house at Nanjing Agricultural University (N 32°2′6.25″, E 118°50′23.47″) from October to December 2018. In the plastic house, plants were grown under 10.50–12.25 h d^−1^ natural light; the maximum light intensity was about 700–800 µmol m^−2^ s^−1^, the average day/night temperature was about 15/10 °C, and the relative humidity was 60%. Both WT and T canola seeds were pregerminated at 28 °C in an incubator for 3 d and then transferred to plastic containers with approximately 50 g of clean sand. One seedling was planted in each container and the seedlings were watered with 1/2 Hoagland’s solution once every 2 d. After 1 month of cultivation, once the fourth leaves had expanded, we transferred the seedlings to hydroponic containers and supplied them with nutrient solutions containing one of three nitrate levels: 3.75 mmol L^−1^ (low N), 7.5 mmol L^−1^ (middle N), and 15 mmol L^−1^ (high N).

### 4.2. Nitrogen Supply and Exogenous ALA Addition

The complete nutrient solution included 6.0 mmol L^−1^ of KH_2_PO_4_, 2.8 mmol L^−1^ of MgSO_4_·7H_2_O, 24 μmol L^−1^ of H_3_BO_3_, 16 μmol L^−1^ of Fe-EDTA (ethylene diaminetetra acetic acid tetrasodium salt), 9 μmol L^−1^ of MnSO_4_, 3.5 μmol L^−1^ of ZnSO_4_, 1 μmol L^−1^ of CuSO_4_, and 0.1 μmol L^−1^ of (NH_4_)Mo_7_O_24_. The low-N solution included 1.25 mmol L^−1^ of Ca(NO_3_)_2_ and 1.25 mmol L^−1^ KNO_3_, the middle-N solution included 1.25 mmol L^−1^ of Ca(NO_3_)_2_ and 5 mmol L^−1^ KNO_3_, and the high-N solution included 5 mmol L^−1^ of Ca(NO_3_)_2_ and 5 mmol L^−1^ KNO_3_. The same concentrations of Ca^2+^ and K^+^ were added to all treatments.

Additionally, we added exogenous ALA at a concentration of 5 mg L^−1^ to the WT canola (exogenous ALA), but not to the ALA-overproducing transgenic canola (endogenous ALA). The control was WT canola without exogenous ALA. All culture solutions were renewed every 3 days. Measurements were taken after 4 weeks of N limitation.

### 4.3. Measurement of Chl Content

We measured the leaf relative Chl content in terms of SPAD values of intact, topmost, fully-expanded leaves using aSPAD-502 Chl meter (Konica Minolta, Osaka, Japan). 

### 4.4. Gas Exchange Analysis

We estimated the photosynthetic gas exchange between 9:00 AM and 11:00 AM on the attached, completely-expanded leaves using a portable gas exchange system (CIRAS-2, PP Systems, Hitchin, UK). Net photosynthetic rate (Pn), stomatal conductance (Gs), transpiration rate (Tr), and intercellular CO_2_ concentration (Ci) were simultaneously recorded at an ambient CO_2_ concentration (approximately 350 µmol mol^−^^1^) and 25 °C, with a relative humidity of 85% and saturating light of 1000 µmol m^−2^ s^−1^.

### 4.5. Simultaneous Measurement of PF and MR Kinetics

We recorded the kinetics of PF and MR simultaneously with a multifunctional plant efficiency analyzer (M-PEA, Hansatech Instrument Ltd., Norfolk, UK) according to the method described by Strasser et al. [34,35]. Briefly, leaves were dark-adapted for at least 30 min before measurement. Then, the dark-adapted leaves were illuminated with an actinic LED light (627 ± 10 nm) at an intensity of 3000 µmol photons m^−2^ s^−1^ per 1 s pulse. During the illumination, the PF and MR kinetics were simultaneously recorded.

We performed a JIP-test of the PF curve according to the method of Strasser et al. [34,35]. The following data from the original measurements were used: fluorescence intensity at 20 µs (O step, Fo), 300 µs (F_K_), 2 ms (J step, F_J_), 30 ms (I step, F_I_), and P step (considered to be maximum fluorescence intensity, Fm). 

The modulated 820-nm reflection signals were represented by the MR_t_/MR_o_ ratio, where MR_t_ is the modulated reflection signal during illumination and MR_o_ is the value at the onset of actinic illumination (taken at 0.7 ms; the first MR measurement). The rapid-decrease phase in MR_t_/MR_o_, from 1 to the minimum value, reflects the PS I oxidation process. The minimum value is a transitory steady state with equal oxidation and rereduction rates in PS I. Subsequently, the slow-increase phase in MR_t_/MR_o_ indicates PS I rereduction. 

### 4.6. Statistical Analysis

We performed statistical analyses with SPSS statistical software (version 22.0, IBM Corp., Armonk, NY, USA) and calculated the means and standard errors of indicated replicates. For multiple comparisons, data among treatments were subject to one-way analysis of variance (ANOVA), and means were compared using Duncan’s tests with significance set at *p* = 0.05. We conducted a two-way ANOVA to compare sources of variation, including ALA, N-level, and ALA × N-level interactions. 

## 5. Conclusions

The results of the study demonstrate that, under various N levels, plants in the ALA treatment groups had higher Chl contents and PS I and PS II activation. Thus, they had higher energy transfer, absorption efficiency, and electron transfer to dark reactions and, hence, higher CO_2_ assimilation rates. Currently, the use of chemical fertilizers on farmland is excessive. Application of exogenous ALA with low-N fertilizer presents a promising strategy for reducing N use while maintaining high photosynthetic capacity in canola and other crops. Moreover, the results for the transgenic line of plants suggest that the manipulation of endogenous ALA is another potential strategy for improving the photosynthetic capacity of crops under various N-supply conditions.

## Supplementary Materials

The following are available online at https://www.mdpi.com/2223-7747/9/11/1419/s1. Figure S1: Changes in (a) the prompt fluorescence (PF) and (b) the variable fluorescence curves (ΔV) of canola leaves under low, middle, and high nitrogen levels. Table S1: Correlation analysis for leaf Chl content, gas exchange, and the considered PF and MR parameters. Table S2: The mean ± SE and statistical tests for the JIP test parameters of each treatment group.
