# Supplementary material for: Photosynthetic Responses of Canola to Exogenous Application or Endogenous Overproduction of 5-Aminolevulinic Acid (ALA) under Various Nitrogen Levels
Source: Plants (Basel). 2020 Oct 23;9(11):1419. doi: 10.3390/plants9111419 (PMC7690814; doi:10.3390/plants9111419)
Supplement: Supplementary file 1 [file plants-09-01419-s001.pdf]

plants

# Supplementary Materials

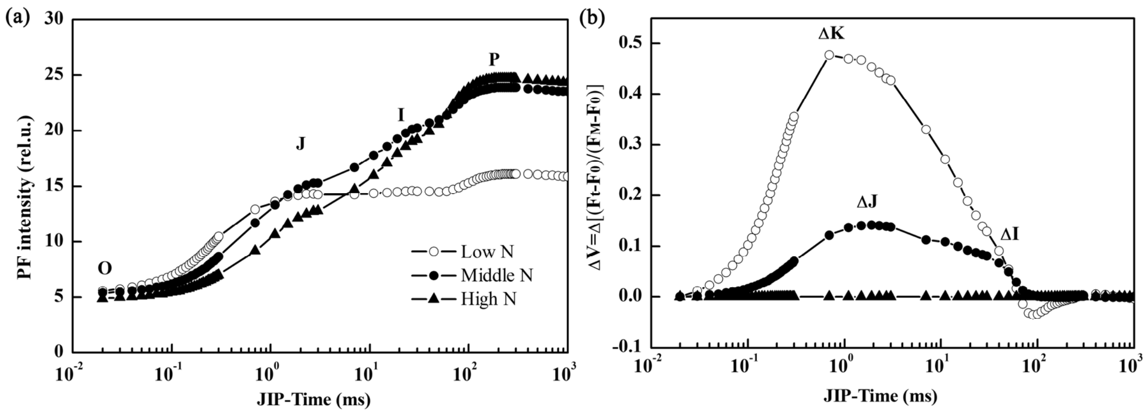


Figure S1. Changes in prompt fluorescence (PF, a) and the variable fluorescence curves (ΔV, b) of canola leaves under low, middle, and high nitrogen levels.

plants

MDPI

Table S1. Correlation analysis for leaf Chl content, gas exchange and the considered PF and MR parameters.

| | Chl content | $P_n$ | Gs | Tr | Ci | $F_o$ | $F_m$ | Fv/ Fm | RC/ $CS_o$ | $M_o$ | RC/ ABS | $ET_o$/ RC | $RE_o$/ RC | $\varphi_{Po}$ | $\psi_{Eo}$ | $\varphi_{Eo}$ | $\delta_{Ro}$ | $\varphi_{Ro}$ | $PI_{ABS}$ | $PI_{total}$ | $V_{ox}$ | $V_{red}$ |
|---|---|---|---|---|---|---|---|---|---|---|---|---|---|---|---|---|---|---|---|---|---|---|
| Chl content | 1.00 | | | | | | | | | | | | | | | | | | | | | |
| $P_n$ | 0.96** | 1.00 | | | | | | | | | | | | | | | | | | | | |
| Gs | 0.91** | 0.92** | 1.00 | | | | | | | | | | | | | | | | | | | |
| Tr | 0.76* | 0.84** | 0.78* | 1.00 | | | | | | | | | | | | | | | | | | |
| Ci | -0.96** | -0.92** | -0.93** | -0.81** | 1.00 | | | | | | | | | | | | | | | | | |
| $F_o$ | -0.60 | -0.68* | -0.39 | -0.43 | 0.49 | 1.00 | | | | | | | | | | | | | | | | |
| $F_m$ | 0.90** | 0.89** | 0.84** | 0.79* | -0.89** | -0.48 | 1.00 | | | | | | | | | | | | | | | |
| $F_v/F_m$ | 0.91** | 0.90** | 0.77* | 0.76* | -0.85** | -0.67* | 0.96** | 1.00 | | | | | | | | | | | | | | |
| $RC/CS_o$ | 0.89** | 0.90** | 0.84** | 0.83** | -0.89** | -0.51 | 0.99** | 0.96** | 1.00 | | | | | | | | | | | | | |
| $M_o$ | -0.90** | -0.91** | -0.80* | -0.78* | 0.86** | 0.62 | -0.97** | -0.99** | -0.98** | 1.00 | | | | | | | | | | | | |
| RC/ABS | 0.91** | 0.95** | 0.81** | 0.80** | -0.87** | -0.73* | 0.94** | 0.98** | 0.96** | -0.98** | 1.00 | | | | | | | | | | | |
| $ET_o/RC$ | 0.91** | 0.91** | 0.78* | 0.74* | -0.85** | -0.68* | 0.96** | 0.99** | 0.96** | -0.99** | 0.98** | 1.00 | | | | | | | | | | |
| $RE_o/RC$ | 0.61 | 0.65 | 0.55 | 0.40 | -0.51 | -0.63 | 0.33 | 0.38 | 0.33 | -0.34 | 0.48 | 0.39 | 1.00 | | | | | | | | | |
| $\varphi_{Po}$ | 0.91** | 0.90** | 0.77* | 0.76* | -0.85** | -0.67* | 0.96** | 1.00 | 0.96** | -0.99** | 0.98** | 0.99** | 0.38 | 1.00 | | | | | | | | |
| $\psi_{Eo}$ | 0.92** | 0.95** | 0.80** | 0.79* | -0.87** | -0.76* | 0.93** | 0.98** | 0.95** | -0.97** | 0.99** | 0.98** | 0.51 | 0.98** | 1.00 | | | | | | | |
| $\varphi_{Eo}$ | 0.92** | 0.95** | 0.80** | 0.78* | -0.87** | -0.77* | 0.93** | 0.98** | 0.94** | -0.96** | 0.99** | 0.98** | 0.53 | 0.98** | 0.99** | 1.00 | | | | | | |
| $\delta_{Ro}$ | -0.58 | -0.54 | -0.49 | -0.54 | 0.58 | 0.24 | -0.79* | -0.79* | -0.80* | 0.82** | -0.70* | -0.79* | 0.24 | -0.79* | -0.68* | -0.66 | 1.00 | | | | | |
| $\varphi_{Ro}$ | 0.93** | 0.96** | 0.83** | 0.75* | -0.85** | -0.76* | 0.82** | 0.86** | 0.82** | -0.84** | 0.91** | 0.86** | 0.79 | 0.86** | 0.92** | 0.93** | -0.39 | 1.00 | | | | |
| $PI_{ABS}$ | 0.90** | 0.94** | 0.77* | 0.75* | -0.84** | -0.84** | 0.79* | 0.85** | 0.80* | -0.82** | 0.92** | 0.86** | 0.75* | 0.85** | 0.93** | 0.94** | -0.38 | 0.97** | 1.00 | | | |
| $PI_{total}$ | 0.88** | 0.92** | 0.76* | 0.76* | -0.81** | -0.82** | 0.74* | 0.80** | 0.75* | -0.77* | 0.87** | 0.80** | 0.81** | 0.80** | 0.89** | 0.90** | -0.29 | 0.97** | 0.99** | 1.00 | | |
| $V_{ox}$ | 0.92** | 0.94** | 0.79* | 0.75* | -0.83** | -0.78* | 0.76* | 0.82** | 0.76* | -0.80* | 0.87** | 0.83** | 0.81** | 0.82** | 0.89** | 0.90** | -0.35 | 0.98** | 0.96** | 0.98** | 1.00 | |
| $V_{red}$ | 0.98** | 0.97** | 0.93** | 0.85** | -0.97** | -0.60 | 0.91** | 0.89** | 0.91** | -0.89** | 0.92** | 0.89** | 0.62 | 0.89** | 0.93** | 0.93** | -0.54 | 0.94** | 0.92** | 0.91** | 0.92** | 1.00 |

*. Correlation is significant at the 0.05 level (2-tailed).

**. Correlation is significant at the 0.01 level (2-tailed).

plants

MDPI

Table S2. The mean ± SE and statistical tests for the JIP-test parameters of each treatment group

| JIP-test parameter | Low N | | | Middle N | | | High N | | |
|---|---|---|---|---|---|---|---|---|---|
| | Control | Exogenous ALA | Endogenous ALA | Control | Exogenous ALA | Endogenous ALA | Control | Exogenous ALA | Endogenous ALA |
| $F_o$ | 5567.67± 49.63 b | 5562.33± 116.88 b | 6143.33± 219.25 a | 5327.67± 163.34 bc | 5227.33± 45.94 bc | 5212.67± 133.37 bc | 4869.00± 162.45 c | 4861.67± 133.14 c | 4850.00± 384.03 c |
| $F_m$ | 16079.67± 1004.98 d | 21756.67± 814.22 c | 23799.33± 1112.10 bc | 23715.67± 1018.08 bc | 26391.33± 959.75 ab | 26900.00± 773.07 a | 24711.33± 967.60 ab | 26566.33± 1057.50 ab | 26190.33± 274.17 ab |
| $F_v/F_m$ | 0.65±0.01 c | 0.74±0.00 b | 0.74±0.03 b | 0.78±0.01 ab | 0.80±0.02 a | 0.81±0.01 a | 0.81±0.01 a | 0.82±0.01 a | 0.82±0.01 a |
| $RC/CS_o$ | 0.29±0.01 f | 0.48±0.02 e | 0.49±0.01 e | 0.55±0.02 d | 0.67±0.02 c | 0.68±0.01 c | 0.68±0.02 bc | 0.73±0.02 a | 0.72±0.01 ab |
| $M_o$ | 1607.08± 65.86 e | 2620.41± 39.01 d | 2945.45± 145.68 c | 2954.89± 63.78 c | 3600.24± 63.94 a | 3582.45± 15.58 a | 3228.70± 117.83 b | 3511.92± 86.38 a | 3454.57± 72.28 ab |
| RC/ABS | 1.87±0.03 a | 0.91±0.02 b | 0.93±0.01 b | 0.70±0.01 c | 0.44±0.01 d | 0.38±0.03 de | 0.44±0.01 d | 0.33±0.02 e | 0.34±0.01 e |
| $ET_o/RC$ | 0.41±0.01 f | 0.62±0.01 e | 0.60±0.01 e | 0.70±0.02 d | 0.74±0.00 c | 0.78±0.02 ab | 0.74±0.02 bc | 0.79±0.01 a | 0.80±0.01 a |
| $RE_o/RC$ | 0.34±0.01 bc | 0.30±0.00 de | 0.30±0.01 de | 0.28±0.00 e | 0.35±0.01 bc | 0.33±0.01 cd | 0.33±0.00 cd | 0.37±0.02 b | 0.44±0.02 a |
| $\varphi_{Po}$ | 0.65±0.01 c | 0.74±0.00 b | 0.74±0.03 b | 0.78±0.01 ab | 0.80±0.02 a | 0.81±0.01 a | 0.81±0.01 a | 0.82±0.01 a | 0.82±0.01 a |
| $\psi_{Eo}$ | 0.18±0.02 e | 0.41±0.01 d | 0.38±0.01 d | 0.48±0.02 c | 0.63±0.03 b | 0.66±0.01 b | 0.65±0.02 b | 0.71±0.01 a | 0.73±0.01 a |
| $\varphi_{Eo}$ | 0.12±0.02 e | 0.31±0.01 d | 0.30±0.01 d | 0.38±0.05 c | 0.50±0.04 b | 0.54±0.00 ab | 0.52±0.01 b | 0.59±0.00 a | 0.59±0.00 a |
| $\delta_{Ro}$ | 0.84±0.01 a | 0.47±0.02 cd | 0.51±0.01 bc | 0.40±0.01 f | 0.46±0.01 de | 0.42±0.00 ef | 0.44±0.01 def | 0.46±0.02 de | 0.53±0.01 b |
| $\varphi_{Ro}$ | 0.10±0.01 e | 0.14±0.01 d | 0.15±0.02 d | 0.15±0.00 d | 0.24±0.01 bc | 0.23±0.00 c | 0.22±0.00 c | 0.27±0.01 b | 0.32±0.01 a |
| $PI_{ABS}$ | 0.12±0.02 f | 0.95±0.01 e | 0.88±0.03 e | 1.84±0.03 d | 5.01±0.08 c | 6.08±0.12 b | 4.79±0.27 c | 7.87±0.03 a | 7.90±0.21 a |
| $PI_{total}$ | 0.55±0.01 c | 0.87±0.01 d | 0.88±0.01 d | 1.24±0.06 d | 4.46±0.03 b | 4.48±0.17 b | 3.66±0.29 c | 6.89±0.47 a | 6.85±0.43 a |

Note: In the same line, values with different letters are significantly different ($P < 0.05$).
